# Arginine Therapy for Lung Diseases

**DOI:** 10.3389/fphar.2021.627503

**Published:** 2021-03-23

**Authors:** Jeremy A. Scott, Harm Maarsingh, Fernando Holguin, Hartmut Grasemann

**Affiliations:** ^1^ Occupational and Environmental Health, Dalla Lana School of Public Health, University of Toronto, Toronto, ON, Canada; ^2^ Department of Pharmaceutical Sciences, Lloyd L. Gregory School of Pharmacy, Palm Beach Atlantic University, West Palm Beach, FL, United States; ^3^ Division of Pulmonary Sciences and Critical Care, University of Colorado, Aurora, CO, United States; ^4^ Division of Respiratory Medicine, Department of Paediatrics and Translational Medicine, Research Institute, The Hospital for Sick Children, Toronto, ON, Canada

**Keywords:** airway hyperresponsiveness, remodeling, chronic obstructive pulmonary desease, cystic fibrosis, Pulmonary hypertension, asthma, asymmetric dimethyl arginine

## Abstract

Nitric oxide (NO) is produced by a family of isoenzymes, nitric oxide synthases (NOSs), which all utilize L-arginine as substrate. The production of NO in the lung and airways can play a number of roles during lung development, regulates airway and vascular smooth muscle tone, and is involved in inflammatory processes and host defense. Altered L-arginine/NO homeostasis, due to the accumulation of endogenous NOS inhibitors and competition for substrate with the arginase enzymes, has been found to play a role in various conditions affecting the lung and in pulmonary diseases, such as asthma, chronic obstructive pulmonary disease (COPD), cystic fibrosis (CF), pulmonary hypertension, and bronchopulmonary dysplasia. Different therapeutic strategies to increase L-arginine levels or bioavailability are currently being explored in pre-clinical and clinical studies. These include supplementation of L-arginine or L-citrulline and inhibition of arginase.

## Introduction

Nitric oxide (NO) is formed by Nitric oxide synthase (NOS) enzymes, in a two-step reaction that uses oxygen and the amino acid, L-arginine, to form NO and L-citrulline. L-Citrulline can be recycled back to L-arginine (Curis et al., 2005), and this L-citrulline recycling has been shown to be particularly important in conditions of reduced substrate availability for NOS, for instance when NOS expression is increased or in the presence of increased endogenous NOS inhibitors (Wu and Morris, 1998; Winnica et al., 2017) Figure 1. NOS monomers, consisting of an oxygenase and a reductase domain, form a homodimer complex at the oxygenase domains that is important for normal NOS functioning. All three of the NOS isozymes can become uncoupled under conditions of low L-arginine availability, low levels of the cofactor tetrahydrobiopterin (BH_4_), increased levels of inhibitors or oxidative stress (Förstermann and Sessa, 2012; Berka et al., 2014). Uncoupled NOS produces superoxide (O_2_^−^) from oxygen which reacts with NO to form peroxynitrite (ONOO^−^), thus potentiating the uncoupling of NOS by lowering the levels of BH_4_, disrupting the NOS homodimer, and oxidizing the zinc-containing core (Münzel et al., 2005; Förstermann and Sessa, 2012). Uncoupling of NOS thus results in a shift of NO production to the formation of peroxynitrite and oxidative stress. Increasing the bioavailability of L-arginine restores NO production and inhibit O_2_^−^ production by NOS.

**FIGURE 1:**
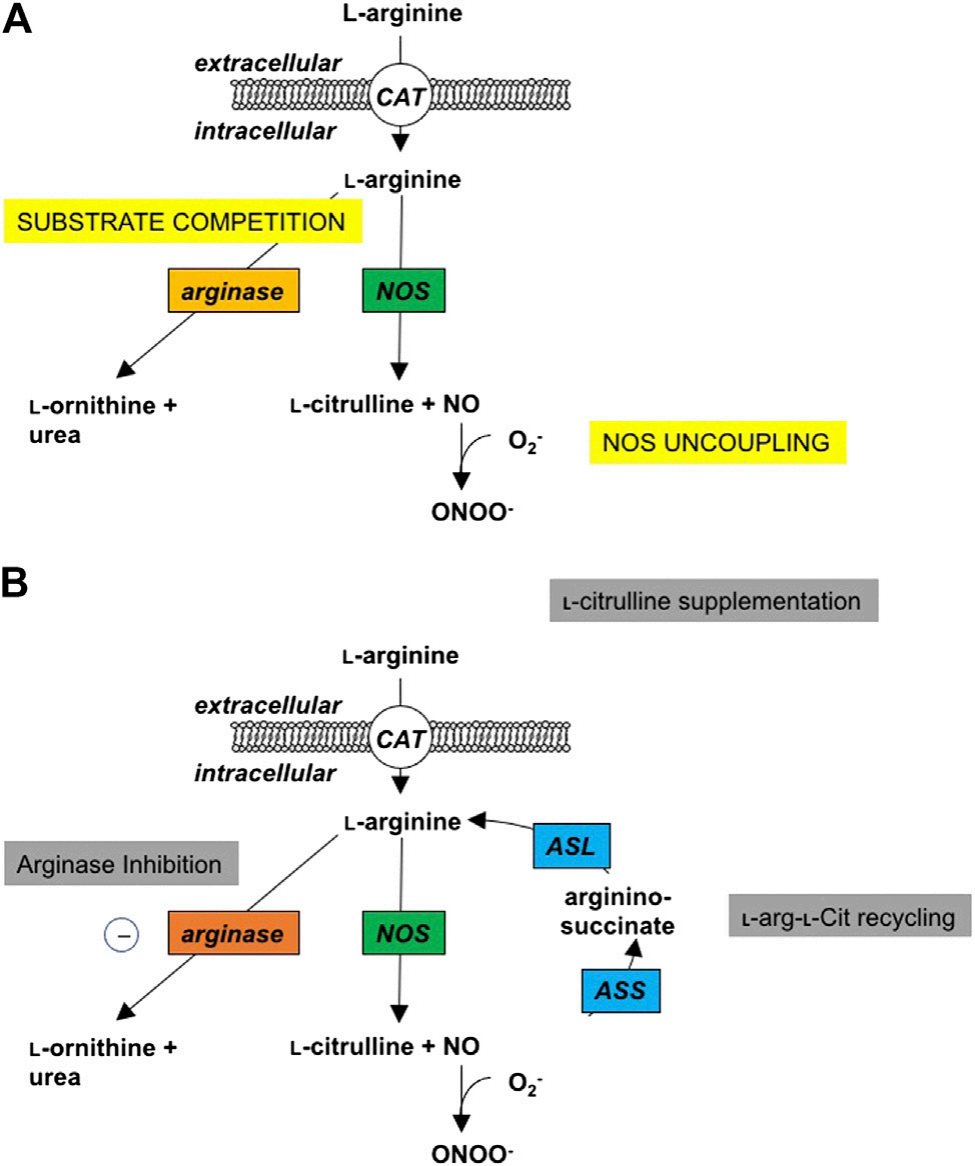
Changes in l-Arginine metabolism in disease and potential interventions. **(A)** Under normal physiologic conditions, cationic amino acid transporters (CAT) transport l-arginine into the cell where it can be metabolized by nitric oxide synthase (NOS) to NO and l-citrulline in a two-step process with Nω-hydroxy-l-arginine (NOHA) as intermediate. Under pathophysiologic conditions, excess induction of the arginase isozymes can lead to increased competition for substrate, thus limiting the l -arginine available for the NOS isozymes, and leading to NOS uncoupling and the production of peroxynitrite. **(B)** As potential sites of intervention, local or systemic administration of arginase inhibitors can increase the cellular bioavailability of l-arginine for the NOS isozymes and improve the production of NO. Supplemental l-citrulline can be recycled to l-arginine by argininosuccinate synthase (ASS) and argininosuccinate lyase (ASL), with argininosuccinate as an intermediate; thus, also improving intracellular bioavailability of l-arginine to improve NO production.

The intracellular activity of all NOS isoenzymes, i.e., the so called constitutively expressed NOS1 (neuronal; nNOS) and NOS3 (endothelial; eNOS) isoforms, as well as the inducible NOS (NOS2; iNOS), is regulated by the availability of substrate L-arginine, which is determined by cellular uptake (Closs et al., 1997), competition with other L-arginine-metabolizing enzymes, particularly the arginase isozymes (arginase I and II) (Wu and Morris, 1998), the presence of endogenous NOS inhibitors, including asymmetric (ADMA) and symmetric dimethylarginine (SDMA) and monomethylarginine (MMA) (Leiper et al., 2007), and L-citrulline/L-arginine recycling (Curis et al., 2005). Methylation of arginine residues in proteins is catalyzed by protein arginine methyltransferases (PRMTs). These methylated arginine derivatives (the endogenous NOS inhibitors ADMA, SDMA and MMA) are liberated as a result of protein degradation. The L-arginine:ADMA ratio (Bode-Böger et al., 2007) provides some insight into NOS activity alterations in pulmonary disease (Holguin et al., 2013; Scott and Grasemann, 2013), with a higher ratio indicating the more normal homeostatic circumstance.

## Increasing Arginine Availability

Since reduced L-arginine availability for NOS has been observed in a number of clinical conditions and diseases, different strategies have been explored to increase L-arginine or the L-arginine:ADMA ratio. L-Arginine is a semi-essential amino acid, which is supplied in the diet and also synthesized from L-citrulline, mainly in the intestinal mucosa (Wu and Morris, 1998). The enzymatic conversion of L-citrulline to L-arginine mainly takes place in the kidney (Curis et al., 2005). In most cells, L-arginine requirements are met primarily by uptake of extracellular L-arginine via specific transport systems (Closs et al., 1997). The efficacy of oral L-arginine to increase L-arginine availability for NOS and subsequently NO formation is limited by a significant first-pass effect. Interestingly, this is not the case for L-citrulline. Oral L-citrulline therefore results in greater increases of circulating L-arginine (via the recycling pathway) and longer circulation time than L-arginine supplementation (Curis et al., 2005; Suzuki et al., 2017).

### Asthma

Elevated fractional exhaled NO (FeNO) in asthmatics is due to activity of NOS2, which is induced during inflammation, in the airways (Ricciardolo et al., 2004). Positive correlations between FeNO, NOS2 expression in airway epithelial and inflammatory cells, airway eosinophilia, and airway hyperresponsiveness (AHR) have been described (Meurs et al., 2003). Reduced L-arginine availability appears to play a key role in allergen-induced AHR, but protein expression of the cationic amino acid transporters CAT1 and CAT2, which facilitate L-arginine uptake, have both been found to be unaltered in lung tissue from asthma subjects (North et al., 2009). However, NOS2 expression in bronchial biopsies from people with asthma was associated with increased presence of nitrotyrosine (Saleh et al., 1998)and ONOO^−^ content correlated with FeNO and AHR suggesting uncoupling of NOS and subsequent ONOO^−^ related airway inflammation in asthma (Saleh et al., 1998). One contributing factor to this could be substrate limitation due to competition for substrate by arginase, which converts L-arginine to L-ornithine and urea. The expression and activity of arginase is increased in lung tissue and airways obtained from various animal models of acute and chronic asthma, as well as in asthmatic patients (Merus et al., 2002; Zimmermann et al., 2003; Maarsingh et al., 2008a; North et al., 2009; Maarsingh et al., 2011); specifically, arginase I expression is upregulated in airway epithelial cells from asthmatics (Zimmermann et al., 2003), and in animal models (North et al., 2009), which may directly affect NO production in the airways. Furthermore, mitochondrial arginase II expression has also been reported to be upregulated in asthma, which may more broadly affect cellular energetics (Xu et al., 2016; Asosingh et al., 2020). Increased serum arginase activity and reduced plasma L-arginine levels have been observed in people experiencing asthma attacks (Morris et al., 2004). The relevance of reduced plasma L-arginine levels to asthma is supported by the finding that allergen-induced AHR in mice was higher in mice that had 50% lower circulating L-arginine levels due to genetic overexpression of arginase I in enterocytes (Cloots et al., 2018). Increased arginase also contributes to NOS impairment by reducing the L-arginine:ADMA ratio (North et al., 2009; Scott et al., 2011; Scott et al., 2014b) and by promoting uncoupling of NOS (Mabalirajan et al., 2010). Increased levels of L-ornithine, the product of arginase activity, could also contribute to the observed NO deficiency in asthma by inhibiting cellular L-arginine uptake (Maarsingh et al., 2008a) and by providing substrate for the production of polyamines, which can also act as potent inhibitors of NOS (North et al., 2013).

Both L-arginine and L-citrulline have been shown to reduce AHR in animal models of allergic asthma (De Boer et al., 2001; Maarsingh et al., 2006; Maarsingh et al., 2008b; Maarsingh et al., 2009a; Maarsingh et al., 2009b; Mabalirajan et al., 2010), and L-arginine alone has also been shown to reduce allergen-induced inflammation in mice (Mabalirajan et al., 2010; Zhang et al., 2015). Oral and inhaled L-arginine increased FeNO in healthy subjects and asthmatic (Kharitonov et al., 1995; Sapienza et al., 1998), but did not affect AHR to histamine (De Gouw et al., 1999). Oral L-arginine supplementation (6–8 g/day) in patients with mild to moderate asthma resulted in an increase in serum L-arginine, ADMA and L-ornithine compared to placebo but had no effects on FeNO, number of exacerbations, or lung function (Kenyon et al., 2011). In a more recent study, oral L-arginine supplementation in severe asthmatics and low FeNO also did not reduce asthma exacerbation rates. However, the higher plasma L-citrulline levels in this study were associated with increased FeNO (Liao et al., 2020).

The effect of the two arginase isozymes on airway inflammation in allergic asthma has also been studied. Genetic ablation of arginase I in myeloid cells did not affect airway inflammation–or AHR–in mouse models of allergic asthma (Niese et al., 2009; Barron et al., 2013). However, a study in female mice demonstrated that deletion of arginase I in myeloid cells attenuated allergen-induced airway inflammation (Cloots et al., 2017), suggesting gender differences in the role of arginase I in regulating inflammation in asthma. Genetic ablation of arginase II actually further increased allergen-induced airway inflammation in mice (Xu et al., 2017; Asosingh et al., 2020), indicative for a protective role of arginase II in airway inflammation. The use of arginase inhibitors that inhibit both arginase I and II has therefore been cautioned. However, in studies in male guinea pigs, arginase inhibitors have shown to decrease (Maarsingh et al., 2008b; Maarsingh et al., 2011) or not alter (van den Berg et al., 2020) allergen-induced airway inflammation. By contrast, arginase inhibition increased allergen-induced airway inflammation in female mice (Ckless et al., 2008). These findings with arginase inhibitors support a potential gender specific role or arginase I in allergic inflammation in asthma.

Obesity is a major co-morbidity in asthma and is associated with poor asthma control. Blood samples from obese asthmatics show increased arginase activity, and lower L-arginine:ADMA ratios, leading to uncoupling of NOS, production of O_2_^−^ as well as oxidative and nitrosative stress (Holguin et al., 2013; Winnica et al., 2017). In a recent clinical trial in obese asthmatics with low FeNO (<30 ppb), oral L-citrulline (15 g/day) supplementation for two weeks increased plasma L-arginine along with the L-arginine:ADMA ratio, increased FeNO, and improved asthma control and lung function, especially in obese females with late-onset asthma (Holguin et al., 2019).

### Chronic Obstructive Pulmonary Disease

Methods for estimating flow-independent airway NO concentrations have suggested that COPD is associated with elevated alveolar NO (Roy et al., 2007). The expression of NOS2 has been shown to be increased in alveolar walls, small airway epithelium, vascular smooth muscle. NOS2 is also increased in sputum macrophages from COPD patients and so is the generation of ONOO^−^ in macrophages and ONOO^−^ content in exhaled breath condensate from COPD patients (Ichinose el al., 2000; Osata et al., 2009). Other studies in COPD patients have shown that FeNO correlated with pre- and post-bronchodilator forced expiratory volume in 1 s (FEV1), and sputum L-ornithine levels correlated with L-arginine and ADMA concentrations. Arginase activity correlated inversely with total NO metabolite (NOx) in sputum, and with pre- and post-bronchodilator FEV1 (Scott et al., 2014a). In a different study, ADMA levels in serum correlated with airway resistance, particularly in patients with poor COPD control (Tajti et al., 2017); further suggesting that ADMA in COPD airways results in a functionally relevant shift of L-arginine metabolism towards the arginase pathway. The functional relevance of this was demonstrated in a guinea model where arginase inhibition shifted the L-ornithine:L-citrulline ratio towards L-citrulline and prevented neutrophilia, mucus hypersecretion and collagen synthesis (Pera et al., 2014). Studies in humans with COPD aiming to increase L-arginine availability for NOS are, to our knowledge, currently lacking. Thus similar to asthma, increasing substrate availability for NOS by arginase inhibition, or supplementation of L-arginine or L-citrulline or a combination thereof, may also be feasible in COPD.

### Cystic Fibrosis

Multiple studies have shown that FeNO is decreased in people with cystic fibrosis (CF) (Grasemann et al., 1997; Elphick et al., 2001), and this may contribute to lower lung function and increased infection risk. Mechanisms contributing to low airway NO in CF may include reduced NOS2 expression (Downey and Elborn, 2000), increased metabolism of NO with the formation of ONOO^−^ (Robbins et al., 2000) and retention in airway secretions (Grasemann et al., 1998) and consumption of NO by denitrifying bacteria (Gaston et al., 2002). In addition, CF airway secretions are rich in neutrophil-derived arginase I, as well as ADMA. These factors all lead to lowered airway L-arginine levels and a state of NO-deficiency (Grasemann et al., 2005b; Grasemann et al., 2006b; Grasemann et al., 2011). A recent study suggested that decreased NO formation and increased protein-arginine methylation may be associated with poor nutritional status in people with CF (Brinkmann et al., 2020). Interestingly, the CFTR modulating drug ivacaftor, which improves CFTR function and clinical outcomes including nutritional status, also increases FeNO in treated CF patients (Grasemann et al., 2015b; Grasemann et al., 2020). Previous studies had shown that increasing L-arginine in CF patients by infusion, inhalation or oral supplementation can increased FeNO, but that only inhaled L-arginine improved lung function (Grasemann et al., 1999; Grasemann et al., 2005a; Grasemann et al., 2006a). Interestingly, a recent study utilizing patient-derived bronchial and nasal cultured epithelial cells, showed that the addition of arginine together with inhibition of arginase activity increased cytosolic NO and enhanced the rescue effect of the CFTR targeting drug ORKAMBI on F508del-CFTR-mediated chloride conductance. The combination of arginine addition with concomitant arginase inhibition also enhanced ORKAMBI-mediated increases in ciliary beat frequency and mucociliary movement. Thus, increasing L-arginine availability for NOS may further increase the efficacy of CFTR modulator therapies (Wu et al., 2019). Another approach to increase L-arginine availability for NOS is through arginase inhibition. Clinical trials are currently underway to study the effect of an oral arginase inhibitor (CB-280) on lung disease in patients with CF (ClinicalTrials.gov: NCT04279769).

### Pulmonary Hypertension

The cause of pulmonary hypertension (PH) is increased vascular resistance in the lung. This often occurs as a consequence of endothelial cell dysfunction, reduced NO, impaired NO-mediated vasodilatory response and/or vascular remodeling (Kaneko et al., 1998; Klinger et al., 2013). The NO deficiency could at least in part be explained by NOS3 uncoupling and increased scavenging of NO due to oxidative stress, and by increased levels of ADMA (Kao et al., 2015; Morris et al., 2005). Increased serum arginase activity and more specifically, endothelial arginase II expression, low plasma L-arginine levels and low l--arginine:ADMA ratios have been described in patients with both primary and secondary PH (Morris et al., 2003; Xu et al., 2004; Morris et al., 2005; Kao et al., 2015). Arginase inhibition has been shown to prevent right ventricular hypertrophy in a guinea pig model of COPD (Pera et al., 2014) and reduce the elevated right ventricular systolic pressure in various animal models of PH (Jiang et al., 2015; Grasemann et al., 2015a; Jung et al., 2017). Arginase inhibition has also been shown to inhibit the hypoxia-induced proliferation of human pulmonary arterial smooth muscle cells (Jiang et al., 2015; Chu et al., 2016), implicating that increased arginase activity could also contribute to vascular remodeling in PH.

Clinical studies in patients with PH have shown positive effects of L-arginine supplementation (Nagaya et al., 2001; Brown et al., 2018). L-Arginine may also be useful in patients with PH and sickle cell disease (Morris et al., 2003; Morris, 2014; Morris, 2017). Supplementation with L-citrulline in newborn infants with chronic PH (Fike et al., 2014) and in patients with idiopathic pulmonary arterial hypertension and Eisenmenger Syndrome (Sharif Kashani et al., 2014) have also been shown to result in improved hemodynamics. Recent studies have also suggested that L-citrulline reduces the risk of postoperative PH in children with congenital heart disease (CHD) undergoing surgery (Smith et al., 2006; Silvera Ruiz et al., 2020).

### Chronic Lung Disease/Bronchopulmonary Dysplasia

Chronic lung disease (CLD) or bronchopulmonary dysplasia (BPD is the major cause of morbidity and mortality in very low birth weight infants (VLBW). BPD is characterized by arrested alveolar development and is complicated by pulmonary hypertension (PH). During lung development, NO has been reported to promote alveolar growth. We have reported changes in the expression of lung arginase throughout the development of experimental BPD/PH, the inhibition of which and/or abrogation leading to improvement in the PH phenotype (Belik et al., 2008; Belik et al., 2009). Supplemental inhaled NO (iNO) also ameliorates the BPD phenotype in experimental models and in some premature infants. Lung parenchymal NO-mediated relaxation is impaired in rat neonates exposed to hyperoxia (Sopi et al., 2007), which could be restored by inhibition of the increased arginase activity (Ali et al., 2012), or with supplementation with L-arginine (Ali et al., 2012) or L-citrulline (Sopi et al., 2012). L-Citrulline supplementation prevents hyperoxia-induced lung injury and PH in newborn rats (Vadivel et al., 2010). A cross-sectional study in neonates reported that L-citrulline levels < 29 μmol/L was associated with BPD/PH (100% sensitivity and 75% specificity); thus, monitoring L-citrulline may be used as a screening tool for BPD/PH (Montgomery et al., 2016). In a clinical study in VLBW infants L-arginine supplementation resulted in survival without CLD was significantly higher in the L-arginine-treated compared with the control group (Polycarpou et al., 2013). As noted previously, the oral bioavailability of L-arginine is limited significantly by the first pass effect, and that this can be circumvented by administration of L-citrulline to engage the L-citrulline/L-arginine recycling pathway. As such, there is currently a trial of oral L-citrulline supplementation in preterm infants that aims to determine the safety, efficacy and dosing for the treatment of BPD/PH (ClinicalTrials.gov Identifier: NCT03649932). Thus, there appears promise in the potential for treatment of BPD/PH through modification of L-arginine bioavailability in the lung.

## Summary

Dysregulation of Larginine/NO metabolism in the lung and airways can contribute to the development of chronic lung diseases, including asthma, COPD, cystic fibrosis, bronchopulmonary dysplasia and pulmonary hypertension. New work aiming to correct for these dysfunctions by increasing L-arginine availability to NOS, focusing on the provision of supplemental L-arginine and/or L-citrulline, as well as inhibition of the competing enzyme, arginase, may lead to improvements in our understanding of the pathogenesis and treatment of these diseases.
